# Aging, Neuromuscular Decline, and the Change in Physiological and Behavioral Complexity of Upper-Limb Movement Dynamics

**DOI:** 10.1155/2012/891218

**Published:** 2012-08-01

**Authors:** S. Morrison, K. M. Newell

**Affiliations:** ^1^School of Physical Therapy, Old Dominion University, Norfolk, VA 23529, USA; ^2^Department of Kinesiology, Pennsylvania State University, State College, PA 16801, USA

## Abstract

Aging is characterized by a general decline in physiological and behavioral function that has been widely interpreted within the context of the loss of complexity hypothesis. In this paper, we examine the relation between aging, neuromuscular function and physiological-behavioral complexity in the arm-hand effector system, specifically with reference to physiological tremor and isometric force production. Experimental findings reveal that the adaptive behavioral consequences of the aging-related functional decline in neurophysiological processes are less pronounced in simple motor tasks which provides support for the proposition that the motor output is influenced by both extrinsic (e.g., task related) and intrinsic (e.g., coordination, weakness) factors. Moreover, the aging-related change in complexity can be bidirectional (increase or decrease) according to the influence of task constraints on the adaptation required of the intrinsic properties of the effector system.

## 1. Introduction

A hallmark feature of aging and the onset of disease is a general decline in physiological function and behavioral capacity [1]. This decline can be manifested in different levels and functions of the biological system, including skeletal muscle [2–4], cardiovascular processes [5, 6], central nervous system activity [7–9], and respiratory function [10], leading to detriments in the behavioral capacity of activities of daily living, including increased tremor, loss of balance control, and a decline in walking ability [5, 6, 11–13]. Understanding the potential reason(s) for decline in function is a challenging undertaking, however, as there are numerous variables that can, either singularly or in combination, affect physiological function in the aging adult. For example, factors related to (but not limited to) biological, behavioral, socioeconomic, nutritional, and/or lifestyle/career choices can all impact on the general process of aging and have implications for physiological function [14–16]. 

The broad range of variables which can negatively affect function in the older adult makes a comprehensive understanding of the direct effect of aging very difficult. In the last decade, the functional deficits in aging have been investigated in the context of changes in the complexity and variability of the output of physiological system(s) [6, 12, 17, 18]. Specifically, the effects of aging are viewed to result in a deficit in physiological function that arises from a progressive “loss of complexity” of the physiological system. This deficit is postulated to arise from a decrease in the functioning number of components or elements of a given system and/or a decrease in the interaction/coupling between components [6, 12, 19]. 

There is not a single definition of a complex system but there is considerable agreement on the properties of complex systems that include (a) many degrees of freedom and interconnections between them and (b) the exhibition of spontaneous self-organization that is adaptive, nonlinear, and dynamic in that it evolves in time, and where order evolves and dissolves without a controller [6, 12, 17, 20]. This theoretical backdrop has led to the experimental emphasis on the time- and frequency-domain structure of variability as opposed to the traditional approach of only considering the dispersion properties of variables through the assumptions about central tendency properties of distributions. Central to this approach has been the use of nonlinear measures of physiological and behavioral time series [20–22]. These tools have revealed changes in complexity with healthy aging and/or age-related diseases like essential tremor, type 2 diabetes, and Parkinson's disease [23–29]. 

## 2. Measuring Physiological Complexity 

Given the inherent complexity of many physiological outputs, there has been a concerted effort to develop appropriate nonlinear tools that can quantify the specific signal of interest [30, 31]. To this end, a variety of measures have been developed and utilized to assess the dynamic properties of specific physiological signals. While a complete review of the differing assessment tools is beyond the scope of this paper, there are certain tools that have been commonly used to assess time series related to physiological processes (a more comprehensive review of the various measures, their use and limitations, is provided by Stergiou and Decker [31, 32] and Bravi et al. [30]). A few of the analyses used include time-frequency analysis, wavelet analysis, recurrence plots, poincare plots, measures of signal entropy (e.g., approximate entropy (ApEn), sample entropy (SampEn), and multiscale entropy (MSE)), correlation dimension, detrended fluctuation analysis (DFA), and Lyapunov exponent [9, 32–43]. 

**Figure 1:**
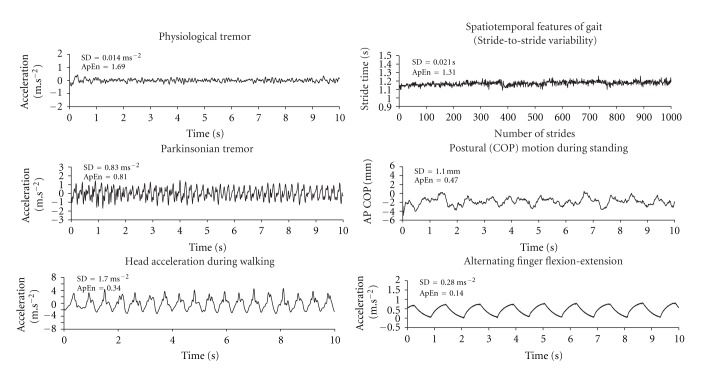
Examples of various physiological signals related to tremor, postural motion, and gait. For each example, an index of the variation in regards to amplitude (SD) and for changes in the variation over the time course of the signal (using approximate entropy (ApEn) analysis) is shown. As this figure illustrates, the more semirhythmical and repeatable signals (e.g., head acceleration, finger motion) were characterized by lower ApEn values, which implies increased regularity (decreased complexity) of the movement signal. Furthermore, the signals that appear more noiselike and irregular (e.g., physiological tremor, stride-to-stride variability) have higher ApEn values implying greater complexity. In contrast, a standard measure of variation (SD) provides little distinction between signals, illustrating that such assessments of variability, by themselves, may be less useful in determining the inherent variability across different movement signals.

Each analytic technique has been designed to assess different aspects of the signal and, in many cases, produces a single outcome measure of the attractor dynamics [18, 30]. One advantage of using such measures to assess complexity is that they are typically dimensionless to the scale of systems and define conditions for dynamic similarities [44]. This allows for the comparison of signal complexity arising from different physiological systems and processes. However, the reliance on any one measure can potentially give a misleading representation of physiological complexity [19]. Therefore, it is recommended that the measurement of physiological complexity be based on multiple measures of system dynamics to increase the sensitivity of complexity assessment under both healthy and pathological conditions [18, 19, 30]. Despite the range of selected measures that can be used to capture the dynamics of a given signal and their limitations, as is highlighted in Figure 1, it is clear that changes in the dynamics of signals are more readily distinguished using nonlinear measures of pattern complexity than the standard dispersion measures (SD, CV) of a variable. 

While there is growing evidence to support the view that aging can be characterized by a general loss of physiological and behavioral complexity, there are findings that challenge the universal nature of the direction of change in complexity with aging. The focus of this paper is to evaluate recent experimental findings as to the effect aging has on neuromuscular function and its relation to changes in physiological and behavioral complexity. While examples for the effects of aging on complexity will be provided for a number of different movement forms, the emphasis is on the neuromuscular function of the arm-hand effector complex given that, for any individual, an optimal degree of hand control is required to perform everyday fine motor skills involving precision, grasping, and/or manipulating small objects [45]. One factor that can negatively impact general hand function is the degree of tremulous oscillations observed during fine motor tasks involving a degree of precision or force control. While these tremors are usually of small amplitude in young adults so that they rarely impact on hand control, these oscillations tend to increase with aging and can severely influence the performance of fine motor skills in older persons [46–50]. Here, we provide an overview of the major aging-related changes in physiological tremor and isometric force production.

## 3. Functional and Structural Adaptations in Skeletal Muscle with Aging

It is widely held that aging is associated with a general decline in skeletal muscle function [3, 4, 51, 52]. One consequence of this decline is that older people lose the capacity to generate task-relevant and/or precise levels of muscle force in the context of action. This decline has been attributed to a loss of overall muscle function [4, 53] and has been associated with changes in a number of mechanisms involving those intrinsic to the muscle and through its neural interface. The specific muscle changes found in the elderly include increases in average muscle force [54, 55], increased motor unit (MU) firing rate variability [56], modulation of MU firing rate [57, 58], altered synchrony between MU recruitment and MU firing rate [51], and reduced sensitivity [59]. Structural changes in the muscle properties associated with aging include a loss (atrophy) of fast twitch motor units and/or switch to slow twitch units (referred to as MU remodeling), altered MU size, and/or a decline in the number of alpha motor neurons within the spinal cord [3, 51, 52, 56, 57, 60]. Consequences of these changes include an overall decrease in muscle cross-sectional area, a reduction in muscle mass, and a decline in strength [4, 16, 61, 62]. 

Generally, the term sarcopenia has been used to describe the loss of muscle mass and low muscle function (strength or performance) associated with aging [63–65]. However, given the diverse range of age-related changes that occur in muscle, which can span basic structural changes to functional changes impacting on the overall “muscle quality,” it has been proposed that the term dynapenia be used to denote those alterations in contractile properties and/or neuromuscular function while sarcopenia be used to describe any age-related loss of muscle mass [15, 16, 66]. It has been proposed that such a distinction would provide a framework for independently assessing those age-related factors which affect muscle mass separately from those variables which impact on neuromuscular function [15]. 

The consequences of these age-related changes in function and mass are that the capacity of skeletal muscle to produce force is compromised in the older adult [4, 62, 67]. Indeed, the muscle responses of older adults are often characterized by prolonged contraction time, an increase in the level of muscle activity for a given level of force production, a decrease in the steadiness at which force can be produced, and a decline in overall muscle force producing capacity [4, 53, 57, 62, 68, 69]. It is clear that there are many types of change that can occur with the normal aging process and that ultimately affect muscle function. 

While aging has been associated with a number of changes at the muscle level, the impact that these changes have on overall movement performance is less well understood. Here we will focus on the relation between aging, neuromuscular function, and physiological-behavioral complexity in the arm-hand effector unit. Aging is associated with a general decline in hand function [46, 47, 70] and there have been numerous investigations of the effect of aging on physiological tremor [49, 50, 71, 72] and isometric force production [47, 57, 69]. This paper will address these changes in physiological tremor and isometric force control within the context of the loss of complexity hypothesis [6, 12, 19], which holds that the process of healthy aging is reflected by a loss of complexity of the respective physiological system. This hypothesis is, in part, derived from the broader construct of dynamical disease [73, 74] and a dynamical systems approach to aging [75, 76], in which physiological systems change due to aberrations in the temporal organization of the evolving dynamics. 

## 4. Physiological Tremor with Aging

**Figure 2:**
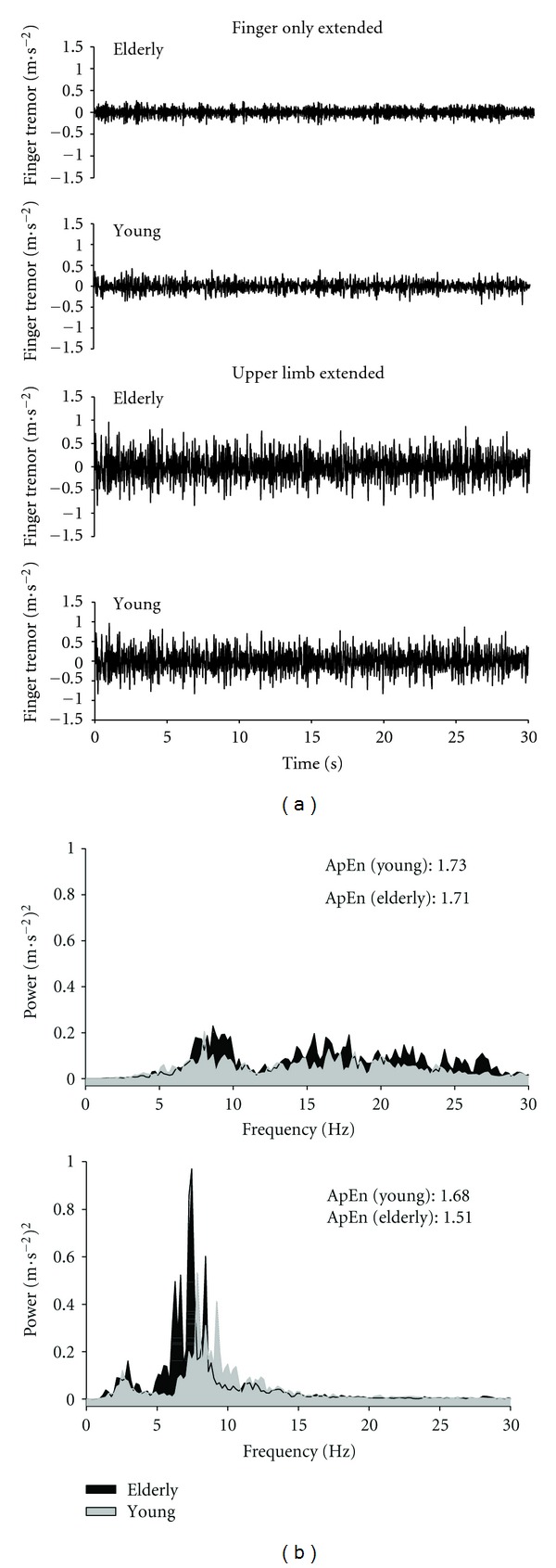
Representative postural acceleration (tremor) traces and power spectral profiles from the index finger of a healthy young and an elderly subject. Traces for each person are shown for conditions where (a) only the finger was extended (the rest of the upper limb was externally supported) and (b) when the entire upper arm was unsupported. Tremor traces were obtained from a single trial for the index finger of each individual. Measures of the degree of regularity (ApEn) of each tremor signal are also shown for each condition. For this analysis, higher values reflect greater complexity within the tremor time series. This example highlights that the age-related differences in finger tremor were only present under conditions where the entire arm was held against gravity.

Physiological tremor is an intrinsic feature of the neuromuscular system reflecting the combined output of multiple oscillatory sources, including the mechanical resonant properties of the specific limb segment, cardiac mechanics, peripheral neural mechanisms (that include contributions from stretch reflexes), and central neural processes [45, 77]. As highlighted in Figure 2, the neural component of a typical tremor signal has most power between 8–12 Hz and represents input from the basal ganglia, inferior olive, deep cerebellar nuclei, thalamus, and, at the spinal cord, alpha motor neurons [45, 78]. One motor symptom linked with aging is an increase in the amplitude of the 8–12 Hz component of physiological tremor, a behavioral consequence that can have negative implications for the ability of an individual to perform everyday fine motor skills. This increase in tremor amplitude is believed to primarily derive from altered central neural output and reflects the more general decline in the functional capacity of the aging neuromuscular system [24, 48–50, 71, 72, 79]. 

Given that physiological tremor is, in part, derived from neuromuscular mechanisms, it is important to isolate the basis of any age-related changes and how they fit within the more general context of our understanding of muscle adaptations with aging. In particular, it is important to determine whether the increases in tremor amplitude are due to a specific decline in aspects of neuromuscular function associated with aging or to a diminished ability of the older neuromuscular system to adapt to more challenging and/or physically demanding task demands. Furthermore, while a long standing view is that tremor tends to increase with aging [49, 71, 72], this position has not been universally supported by contemporary experimental research [48, 50]. For example, several studies have observed no age-related increase in tremor amplitude and only subtle changes in the frequency of the 8–12 Hz neural tremor peak [41, 50, 80]. The significance of these findings cannot be understated, because if the established changes in muscle physiology with aging do not translate to increases in physiological tremor, then the reasons for this dissociation remain to be fully elucidated. One suggestion as to why these studies have not reported aging-related increase in physiological tremor is that the changes may only be detectable in the oldest-old members of the population (e.g., for persons aged 80 years or older [41]). An alternative reason for the lack of aging-related change in tremor amplitude may be due to the conditions or tasks under which physiological tremor is assessed [81]. 

In the majority of studies of age-tremor effects, the common practice has been to limit the assessment of these oscillations to the tremor within a single (usually the finger) segment [41, 50, 79, 82]. In this situation, the more proximal segments are supported externally and do not contribute to the oscillations seen distally. This experimental approach has been employed in an effort to tease out and isolate the specific age-related adaptations in muscle physiology since the action is restricted to a single segment, joint, and/or muscle group. However, while this approach allows a more direct evaluation of the responses of an individual muscle, it has been argued that this protocol is somewhat artificial, since there are few (if any) instances during everyday tasks where persons are required to perform a functional, goal-directed action involving a single muscle and/or segment. Conversely, under more real world conditions where individuals need to maintain the postural position of the entire limb, tremor is rarely localized to a single segment. Consequently, while single joint movements provide insight as to the intrinsic function of a specific, isolated muscle, it remains an open question as to what these findings reveal about the challenges the aging neuromuscular system faces when performing everyday actions involving multiple limb segments. 

An alternative experimental approach has been to examine postural tremor when the entire upper arm is unsupported. This protocol provides a more realistic evaluation of the tremor responses apparent in the performance of everyday actions and insight into differences due to aging or disease [24, 83–86]. Tasks of this nature are inherently more challenging as there is now a more substantive strength requirement (e.g., to sustain limb position against gravity) and the need to adaptively compensate for the tremor in multiple segments so they do not all sequentially magnify the oscillations at the more distal segments [72, 87]. Given that these tasks place increased demands for control on the neuromotor system, it has been suggested that examining tremor from different segments when the entire arm is unsupported may prove to be useful in discerning between neurologically healthy and clinical populations [83, 84, 86]. 

**Figure 3:**
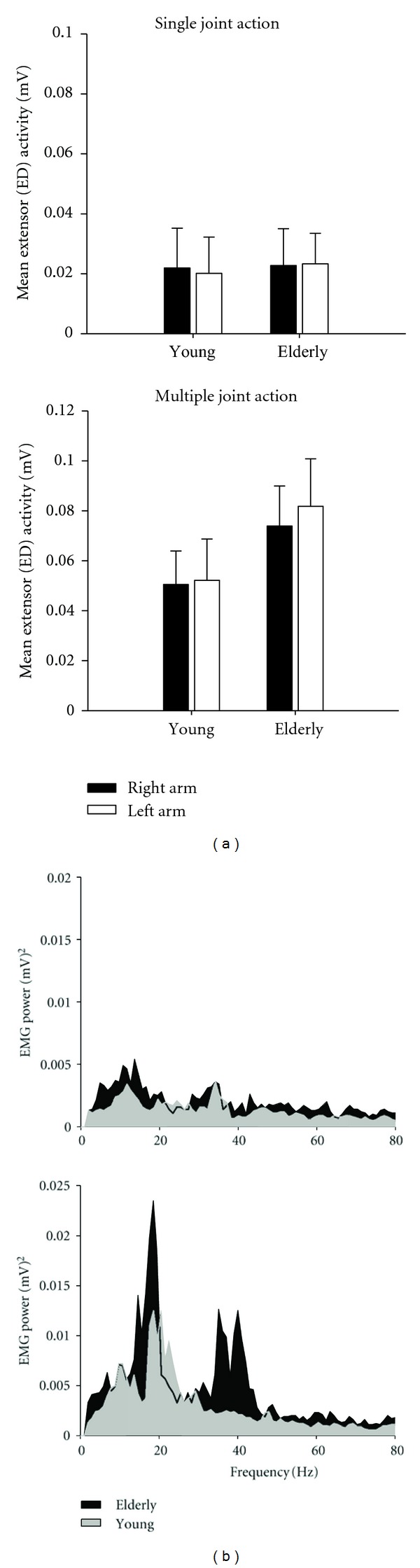
Overall changes in mean RMS EMG activity from the extensor muscles of the forearm and examples of the power spectral profiles for a healthy young and elderly individual. Traces for each person are shown for conditions where only the finger was extended (the rest of the upper limb was externally supported) and when the entire upper arm was unsupported. As with Figure 2, any age-related differences in muscle activity were only seen under physically demanding task conditions.

Indeed, when this approach has been adopted, very clear and notable differences in tremor are found as a function of normal aging [24, 72], Parkinson's disease [27, 85, 86, 88, 89], and multiple sclerosis [83]. The typical pattern of results for this type of approach, as shown in Figures 2 and 3, is that older persons tend to exhibit greater hand and finger tremor coupled with increased muscle activity in the forearm extensors when required to hold the entire arm against gravity, compared to the EMG/tremor responses when only the finger is extended (the other segments were supported externally). For the healthy elderly individuals, where tremor increases were reported, these were limited to the more distal segments only (e.g., the hand and finger), with no notable changes in the tremor from the forearm or upper arm [24, 72]. Further, the age-related increases appear to be exacerbated when the older person performs the task in a standing position compared to sitting [24], which supports the view that relatively simple postural adjustments can also influence tremor dynamics [87]. In both situations, however, the tremor increase is primarily restricted to the neural 8–12 Hz component and related muscle activity, indicating that changes in the output of those central neural processes underlying tremor genesis were responsible for the aging-related differences. 

These contrasting findings on single versus multiple segment tremor invite the question as to the relative difficulty of movements performed about a single joint/segment. For comparison, the amplitude of physiological tremor observed from the finger under single segment conditions has been reported to be within the range of 1–3 mm [90], whereas, for tasks requiring individuals to hold their entire arm up against gravity, oscillations of the order of 10–20 mm have been reported for the index finger [24, 72]. If one considers the task goal during these actions was to minimize limb postural motion (tremor), there is a higher degree of difficulty in controlling the muscles about an entire limb to minimize oscillations (and hence the greatest potential for actually being able to reduce tremor) under conditions where the entire arm must be coordinated and controlled, and not just the oscillations in a single distal segment. However, optimal performance for task of this nature is not simply the result of increasing muscle activity. Previous research has demonstrated that when subjects actively cocontracted the muscles of the arm to stiffen the arm, the degree of tremor at the finger increased significantly [91]. Consequently, individuals need to find a balance between required levels of muscle activity to hold the limb against gravity while also be able to achieve a necessary degree of control to ensure limb oscillations are kept to a minimum. It is likely that a combination of a loss of muscle function and control (dynapenia) and muscle strength (sacropenia) in older adults contributes significantly to their increased tremor responses. 

The age-related adaptive changes in the tremor tasks show that the aging neuromuscular system is less able to adapt to the constraints of performing more challenging and/or physically demanding everyday tasks. Under these situations, the capacity of the older person is stressed more and so the effects of the changes at the individual muscle level are aggregated in some way that is reflected by an increase in physiological tremor. In comparison to single-joint tremor actions, older participants find multiple segment tremor tasks more demanding and so the increased tremor reflects the greater demands of holding the entire limb unsupported. Furthermore, the selective changes in EMG activity and the 8–12 Hz neural component of tremor for this type of action support the position that increased neuromuscular drive generated in response to the more challenging task conditions is a contributing factor to revealing the aging-related increases in oscillatory outputs [51, 58]. 

## 5. Aging Changes in Physiological Complexity of Tremor

In addition to the challenges about the theoretical relevance of examining tremor in single joint versus multiple joint postural tasks, there is still, as noted previously, ambiguity as to whether tremor variability actually increases with the process of aging. In an effort to provide greater insight as to the effects of aging and/or disease on physiological and behavioral processes, there has been an evaluation of the spatial/temporal pattern of the given tremor signal using measures of complexity [6, 12, 17]. 

This experimental approach to aging is based on the proposition that there is a deficit in physiological function that results from a progressive loss of complexity (i.e., dynamic variability) of the physiological system. This deficit has been phrased “loss of complexity” and is postulated to arise from the general decrease in the number of elements of a given system and/or decrease in the interaction/coupling between control processes [6, 12, 19]. Given the complex nature of the oscillatory output that is physiological tremor, it is natural that the theoretical perspective of complexity has been drawn on to examine the questions of the dynamics of aging and disease with associated measures that are beyond the standard dispersion indices of variability (e.g., standard deviation). 

 Central to this approach has been the use of dynamical nonlinear measures of a physiological and behavioral time series [20–22]. These tools have revealed changes in complexity with healthy aging and/or age-related diseases including essential tremor and Parkinson's disease [23–27, 85]. One of the more commonly used measures has been approximate entropy (ApEn), which has also been employed to assess complexity changes for a variety of physiological related signals including hormone secretion, isometric force outputs, muscle activity, heart rate, postural motion, and gait [37–40, 92, 93]. ApEn measures the probability that runs of patterns that are close for m observations remain close on the next (*m* + 1) incremental comparisons. This analysis produces a single value (range of 0–2) with higher values reflecting greater irregularity while lower values represent a greater repeatability or higher regularity in the time series. For example, using approximate entropy (ApEn) measures, Sturman and associates [41] reported that there was an increase in the time-dependent structure of physiological tremor with advanced age, despite there being no differences in tremor amplitude between the respective age groups. Other studies have reported similar age-related differences in physiological tremor using the same analyses [24, 72, 81]. Interestingly, Hong et al. [81] conducted a study to examine whether there were any age-related differences for tremor in the frontal and transverse planes of motion. While no aging-related effects were observed for tremor in the vertical direction, changes in the tremor ApEn values for motion in the mediolateral axis between the young and older adults were reported. 

Together, these results support the view that aging and disease can be reflected by a change in the time-dependent pattern or structure of the specific tremor signal output. This result, combined with the lack of any age differences in signal regularity during the finger only conditions (see Figure 2), is consistent with the proposition that the neuromuscular system of older individuals is typically not challenged enough under the single segment condition to necessarily reveal any appreciable change in the system dynamics. The added strength and coordination demands placed on the older individual of having to hold their upper arm against gravity and minimize tremor support the general premise of the loss of complexity hypothesis. 

While it has been proposed that the general aging process is accompanied by a decrease in physiological complexity [6, 12], it is important to note that, with regard to physiological tremor, the results of previous studies do not universally support this perspective since there appears to be no consistent difference in the structure of tremor signal under single segment conditions. The fact that several studies have either reported no change or a decline in physiological complexity for tremor signal dynamics in older people lends support to the proposition that the hypothesis of a unidirectional nature of the loss of complexity hypotheses is too narrow [18]. A contrasting perspective is that there can be an increase or decrease in a given signal's pattern over time depending on the interaction between components of the biological system and the inherent task dynamics [18]

There is empirical evidence to support the position that the aging-related changes in signal complexity can be bi-directional. In a recent study [85], it was reported that the physiological tremor of older persons with Parkinson's disease (PD) exhibited a loss of complexity compared to the healthy individuals of a similar age. However, the whole body motion (COP) of these same PD individuals was characterized by an *increase* in signal complexity when compared to the healthy elderly. This reciprocal pattern of change in these oscillatory signals within the same subjects supports the bi-directional perspective on changes in complexity with aging. Similarly, Hong et al. [81] reported that the only significant aging-related change in finger tremor was for side-to-side motion, while tremor in the vertical plane exhibited no difference between young, old (60–65 yrs), and older-old (70–75 yrs) individuals. It would seem that a strong contributor to the observed age differences in signal complexity is the older individual's need to increase their neuromuscular output so as to realize the specific demands of the task being performed [17]. 

 The bi-directional hypothesis for the change of complexity in the movement dynamics with aging is based on the framework that the confluence of organismic, environmental, and task constraints channels the coordination and control of the system degrees of freedom [17, 18, 94]. In this view, the aging and loss of complexity effect will hold when an increase in the dimension of behavior is required from the intrinsic dynamics to realize the task demands. And the bi-directional effect of an increment in complexity will be prevalent when the confluence of constraints channels a reduction in the functional degrees of freedom of the system. As we note later, a good example of this bi-directional hypothesis is in isometric force control [94] where aging leads to a loss of complexity in the control of a constant force level (where better performance is realized by increasing the functional degrees of freedom) and an increment in complexity in a sine wave force tacking (where better performance is realized by reducing the degrees of freedom of the intrinsic dynamics). In this framework, the aging effect is more generally a loss of adaptation of the functional degrees of freedom rather than universally loss of complexity.

## 6. Isometric Force Production with Aging

**Figure 4:**
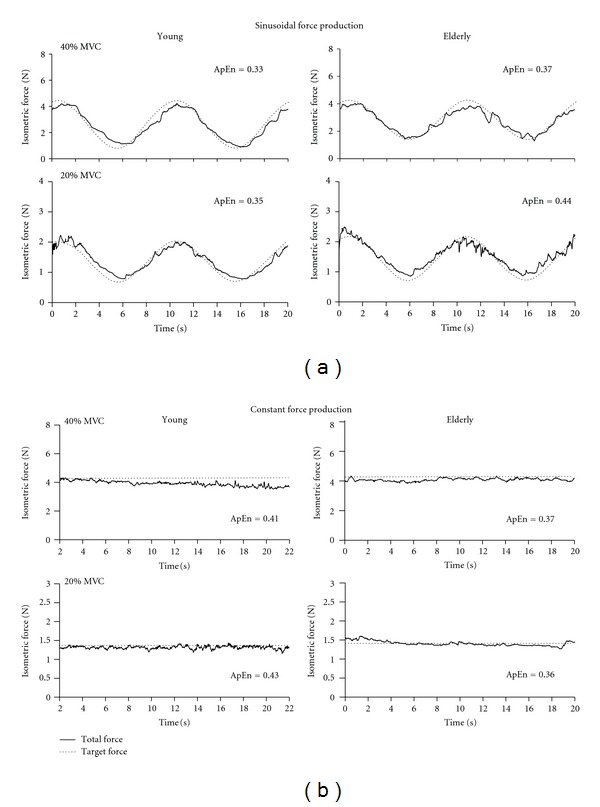
Representative examples of isometric force production trace (40% and 20% MVC) for a single young and older person. Examples are shown as individuals tracked a sinusoidal and constant target force. All traces were attained from a single subject during a single trial within each condition.

In grasping actions, individuals need to produce a certain degree of isometric force in order to hold and/or manipulate a given object [95, 96]. When producing this action, one consequence is the production of small fluctuations in the force output, that have been referred to as reflecting force steadiness or isometric force tremor [68, 97]. Healthy older individuals, in comparison to young adults, often exhibit reduced control in force production, as quantified by an increase in these fluctuations [59, 98, 99]. Interestingly, this age-related decline in force producing capacity has typically been interpreted to reflect changes in motor unit (MU) control and sensorimotor function rather than in terms of more macro the constraints such as muscle strength per se. The consequence of these changes is that elderly adults exhibit greater targeting error and isometric force variability. As illustrated in Figure 4, both of these features of variability tend to be more pronounced when producing lower maximum voluntary contraction (MVC) forces in comparison to higher maximal forces [57, 68, 100–102] and during force tracking tasks where a sinusoidal target is displayed in comparison to a constant force target [103]. 

Given the prevailing view that overall muscle strength declines with aging [3, 4, 13, 67], the finding that it is more difficult to produce accurate levels of force output at lower MVC levels seems somewhat counterintuitive. If a decline in force producing capacity was to be the principal mitigating factor in the loss of muscle function in the older adult, then it would be predicted that producing higher forces would be more difficult. What these studies demonstrate is that any age-related changes in movement ability are not merely the product of alteration within the older muscle itself. Indeed, similar to the findings shown previously for physiological tremor tasks, it would appear that the effects of aging are amplified under more challenging actions (e.g., sinusoidal versus constant force production). 

However, one important distinction can be made regarding the age-related changes in both physiological and isometric tremor. For physiological tremor, the argument often made is that the increased tremor amplitude reported where the entire arm is held against gravity primarily reflects the diminished strength of the older person. However, the same argument cannot be made for isometric force tremor, since here the greatest difference is in performing tasks of lower force levels. Under isometric conditions, the suggested reason(s) for the age-related differences in performance at lower force levels typically draws on the manner in which the neuromuscular system modulates MU recruitment and firing rate(s) in order to accurately grade force output [51, 94, 104]. Within this context, it would appear that the age-related variation in isometric force production dynamics is driven more by task-specific control and coordination constraints rather than the ability to produce (and sustain) high absolute force levels [69]. 

Further support for the notion that chronological age *per se* does not always drive the changes seen in force production comes from a study by Sosnoff and Newell [105]. They reported that when differences in the maximal voluntary contraction (MVC) force of young and older individuals were controlled for, there was no performance difference in terms of the isometric force variability between age groups. From these findings it was argued that chronological age is not, by itself, a sufficient indicator of the decline in isometric force control, but rather that the relative degree of weakness, irrespective of age, is a more appropriate biological index. This result is of some importance since it would indicate that any age-related declines in isometric force control maybe more a function of inactivity, and so is modifiable by training, rather than simply the inevitable process of decline associated with chronological aging. Indeed, subsequent studies have demonstrated that improvements in isometric force control can be elicited with specific exercise interventions [55, 106].

## 7. Aging and Complexity in Isometric Force Control

**Figure 5:**
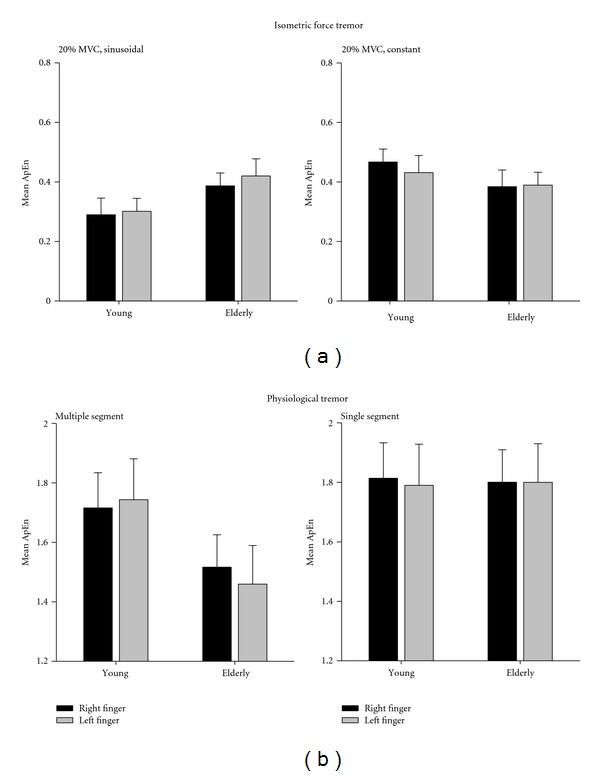
Age-related differences in approximate entropy (ApEn) measures for isometric force (a) and postural tremor (b) tasks. Changes in ApEn across the young and older individuals are shown for both the multiple segment and single-segment tremor tasks. In addition, the age-related differences during two isometric actions (20% MVC performed under sinusoidal tracking and constant force conditions) are also shown. This figure illustrates that the tremor signal tends to be less complex (lower ApEn) during multiple-segment tremor tasks and the 20% MVC constant force producing actions. However, for the 20% MVC sinusoidal funder isometric force task, the resultant signal for the older adults is more complex (higher ApEn) in comparison to the younger adults. Error bars represent one standard error of the mean.

As with the assessment of physiological tremor, additional insight as the any age-related changes in force production has been reported when measures of complexity have been applied to the time series. In addition to the straightforward assessments of changes in force variability or targeting error, many studies have reported that the age-related differences in force control extend to differences in the frequency profile of their force output, the pattern of regularity (based upon changes in ApEn and SampEn), and, where multiple digits are employed, changes in the coupling relations between these effectors [100, 101, 103, 105, 107, 108]. When reviewing the resultant force signals, it is interesting to compare the age-related differences in signal complexity for isometric actions with the responses generated for physiological tremor tasks. As shown in Figure 5, the force response from older adults is highlighted by an increase in complexity (increased ApEn) in comparison to younger individuals when performing more challenging isometric task (e.g., 20% MVC, sinusoidal tracking). However, during more demanding postural tremor tasks (e.g., whole arm extended, see Figure 2), the tremor output from the older adult was characterized by a decline in complexity (lower ApEn). 

It would appear that, as with the discussion of the effect of aging on physiological tremor, the changes in the isometric force producing ability of older person can only in part be explained by the loss of complexity hypothesis [6, 12, 19]. While the force signal for the older adult typically exhibits increased complexity under more challenging task conditions, this pattern is not consistently prevalent across less challenging force levels or when different effectors are utilized to perform the task [57, 69, 105, 107]. This invites the interpretation that any age differences in complexity of the signal output are more a function of the interaction between extrinsic (task) and intrinsic (sacropenia, dynapenia) factors rather than biological age being the single driving factor. Consequently, the pattern of changes in the biological signal are not consistent with the view that aging is reflected by an overall loss of complexity [18]. Rather, the dynamics in the isometric force task reflect the confluence of constraints including those of the aging individual, the task constraints, and those of the environment. 

## 8. Summary

With aging, there is a general decline in the physiological function that is often manifested by specific changes in the functional and structural properties of skeletal muscle [2–4, 51]. This decline in functional capacity of a given system has been increasingly viewed within the context of the loss of complexity hypothesis [6, 12]. While these changes alter the capacity of the individual muscle to respond, it is not clear to what degree these changes have a universal impact on an individual's behavioral movement performance in physical activity. 

In the current paper, we examined the relation between aging, neuromuscular function, and physiological-behavioral complexity, specifically with reference to physiological tremor and isometric force production. These two motor outputs were selected since they both derive primarily from neuromuscular mechanisms, and the ability to control and minimize these oscillatory outputs is essential for the performance of many activities of daily living (ADL's) which contain a fine motor skill component. The examination of age-related changes in these motor processes would therefore provide greater understanding of the relation between muscle adaptations and chronological age. A central point to emerge is that there is no single pattern to the changes seen in physiological and isometric force tremor in older adults. Rather, it would appear that the specific alterations in the given motor outputs reflect a myriad of extrinsic (task related) and intrinsic (muscle weakness, loss of coordination) constraints that are unlikely to be all the direct result of the process of aging. Consequently, it is argued that any amplitude or structural changes observed in physiological and force tremor amplitude reflect the diminished ability of the older neuromuscular system to adapt to differing task demands. 

Finally, the findings of this body of research do not universally support the unidirectional interpretation that aging is associated with a loss of physiological and behavioral complexity. Instead, the variable pattern of change in complexity observed across both physiological and isometric tremor forms in older adults supports the broader view that age-related changes in physiological complexity are bi-directional, depending to a large degree on the constraints to action. Thus, the adaptive responses of the upper limb movement dynamics studied do not simply reflect the impact aging has on motor function, in that they also depend on the task-specific requirements of the given action. Our synthesis provides further evidence that chronological age should be viewed as just an entry variable into the problem of the study of aging and not, by itself, an inevitable causal factor in neuromuscular decline and the change in physiological and behavioral complexity.
